# Genome-Wide Profiling of the ACTIN Gene Family and Its Implications for Agronomic Traits in *Brassica napus*: A Bioinformatics Study

**DOI:** 10.3390/ijms251910752

**Published:** 2024-10-06

**Authors:** Shengli Yao, Jiayu Peng, Ming Hu, Qing Zhou, Xiuju Zhao

**Affiliations:** 1 College of Life Science and Technology, Wuhan Polytechnic University, Wuhan 430023, China; 2 Horticultural Crop Biology and Germplasm Enhancement, College of Horticulture, South China Agricultural University, Guangzhou 510642, China

**Keywords:** *Brassica napus*, the *ACTIN* family, agronomic traits, association mapping analysis

## Abstract

ACTINs are key structural proteins in plants, which form the actin cytoskeleton and are engaged in numerous routine cellular processes. Meanwhile, *ACTIN*, recognized as a housekeeping gene, has not yet been thoroughly investigated in *Brassica napus*. The current research has led to the detection of 69 actin genes in *B. napus*, which were organized into six distinct subfamilies on the basis of phylogenetic relationships. Functional enrichment analysis, along with the construction of protein interaction networks, suggested that *BnACTIN*s play roles in Preserving cell morphology and facilitating cytoplasmic movement, plant development, and adaptive responses to environmental stress. Moreover, the *BnACTIN* genes presented a wide range of expression levels among different tissues, whereas the majority experienced a substantial increase in expression when subjected to various abiotic stresses, demonstrating a pronounced sensitivity to abiotic factors. Furthermore, association mapping analysis indicated that some *BnACTIN*s potentially affected certain key agronomic traits. Overall, our research deepens the knowledge of *BnACTIN* genes, promotes the cultivation of improved *B. napus* strains, and lays the groundwork for subsequent functional research.

## 1. Introduction

ACTIN protein is highly conserved in eukaryotes, which participate in cytoskeletal formation and various cellular processes that have a significant influence on plant growth and development [1,2]. The plant actin cytoskeleton is irreplaceable for its role in the connection of proteomic functions to cellular life activities [3,4,5]. A multitude of cellular processes in plant cells relies on the cytoplasmic actin cytoskeleton, which is key to cell fission and expansion regulation, propelling the flow of cytoplasm, nurturing growth at the tips, forming and sustaining cell contours and directional properties and orchestrating organelle transport and relocation [6,7,8]. Moreover, the cytoskeleton frequently acts as a mediator of stress response in plants [9,10]. For instance, *ACTIN*s may be employed as candidate genes to fortify sweet potato against abiotic stress [11]. ACTIN filaments are crucial in stress-induced signaling pathways, serving as both direct targets and signal transducers [12]. ACTIN can exist in the form of monomeric G-actin or as a part of filamentous F-actin. G-ACTIN represents the soluble, globular monomeric form of ACTIN, capable of polymerizing to create F-ACTIN, which then constitutes the cytoskeleton and the contractile machinery of muscle cells, driving cell movement and muscle contractions [13,14]. Additionally, ACTIN stands as a highly conserved protein with a molecular mass of about 42 kDa and consists of a polypeptide chain made up of 375 amino acids. At least six types of actin have been isolated in mammalian and avian cells, four of which are called alpha-actins, specifically found in skeletal muscle, cardiac muscle, vascular smooth muscle, and intestinal smooth muscle, respectively; the other two are beta-actin and gamma-actin, which are present in all muscle cells and the cytoplasm of non-muscle cells. Nevertheless, multiple genes in plants encode ACTINs, leading to the existence of various *ACTIN* isoforms [15,16,17]. In order to accommodate various functions, multiple *ACTIN* isoforms with diverse functions are coexpressed in different eukaryotic organisms [15,18]. ACTIN serves as a key constituent of the organelle skeleton, crucial for the life-sustaining functions of plants, and is frequently used as an internal reference gene owing to its consistent expression across diverse cellular physiological conditions [19,20].

In the past, research on the structural and functional aspects of actin genes has been chiefly concerned with higher eukaryotes. In terrestrial plants and animals, actin genes are generally the product of a diverse gene family, a result of gene duplication and subsequent diversification [21]. So far, actin genes have been identified in several plants at the genome-wide level, including 20 in *Arabidopsis thaliana*, 16 in grape, 22 in rice, 18 in poplar, and 30 in sweet potato, following the rapid development of high-throughput sequencing [4,15,22,23]. Multiple distinct actin isoforms have been recognized in these species, all encoded by separate genes. In *Arabidopsis thaliana*, *ACTIN*s are categorized into six subfamilies, the majority of which display tissue-specific expression patterns [24]. Moreover, according to their phylogenetic relationships and expression profiles, the eight genes can be divided into two main categories: one vegetative class, predominantly expressed in leaves, stems, roots, petals, and sepals, and a reproductive class, strongly expressed in pollen, ovules, and embryonic tissues. In the case of *AtACT1* and *AtACT3*, their expression is observed in all organ primordia and mature pollen. *AtACT4* and *AtACT12* are specific to mature pollen and young vascular tissue, whereas *AtACT11* is highly expressed in the ovule, embryo, and endosperm [25]. Moreover, *AtACT2* and *AtACT8* are active in a wide range of vegetative tissues [24,26,27], while the misexpression of *AtACT1* led to stunted plant growth and changes in organ shape [28]. Furthermore, the double mutation of vegetative genes *AtACT2* and *AtACT8* (*act2/8*) was associated with larger leaf size and increased ploidy in mature leaves, while Suppressing *AtACT2* and *AtACT8* caused roots to be completely devoid of hairs [29,30]. In addition, a recent investigation reported that the cotton (*Gossypium hirsutum*) possesses 16 actin gene family members, exhibiting differential expression across various organs, which was determined through real-time PCR and RNA gel blot analysis [31].

Allotetraploid *Brassica napus* (A_n_A_n_C_n_C_n_, 2*n* = 38) is one of the crucial oil crops, which possesses a high economic and appreciatory value [32,33,34]. *B. napus* evolved from the hybridization event between *Brassica rapa* (A_r_A_r_, 2*n* = 20) and *Brassica oleracea* (C_o_C_o_, 2*n* = 18), accompanied by chromosome doubling nearly 7500 years back [35,36,37]. *Brassica* species, members of the *Brassica* genus and the Brassicaceae family, serve as an excellent model for investigating the evolution of polyploid genomes, as well as the processes of gene duplication, gene loss, gene emergence, and modification of gene functions [32,38,39]. Beyond supplying high-quality oil that contains minimal saturated fatty acids and cholesterol while being rich in microelements, it also contributes to the production of animal feed and the generation of biodiesel [40]. In the context of the rapid progress in high-throughput genomic sequencing, the accessibility of genome sequences for *B. napus*, *B. oleracea*, and *B. rapa* has presented a unique chance to discover and define pivotal genes on a genomic scale.

Up to now, although the biological roles and regulatory dynamics of *ACTIN*s have been investigated in several higher plants, they have yet to be understood in *B. napus*. In this research, a total of 69, 37, and 31 *ACTIN* genes were identified in allotetraploid *B. napus* and its diploid progenitors (*B. oleracea*, *B. rapa*), respectively. Additionally, this study conducted a comprehensive analysis of *ACTIN*s in rapeseed, encompassing molecular properties, evolutionary relationships, expression patterns, and possible influences on key agronomic traits. In a word, the findings of the study provided a deeper insight into *BnACTIN* genes, establishing a foundation for forthcoming investigations into gene function and genetic improvement.

## 2. Results

### 2.1. Genome-Wide Characterization of ACTIN Genes in B. napus and Its Diploid Progenitors

The ACTIN domain annotation file (PF00022) sourced from the Pfam database was employed as a query to search for UBC family members within the *B. napus* protein dataset. Moreover, assumed BnACTIN peptides, which exhibited matches to AtACTINs through BLAST, underwent additional verification for ACTIN domain existence by searching in the Pfam, SMART, and CDD repositories. A total of 69 BnACTIN family members were discovered within the *B. napus* proteome, which was listed in Supplementary Table S1 for details. *BnACTIN* full-length transcripts vary in length from 825 bp to 3296 bp, with the corresponding protein sequences extending from 275 aa to 993 aa. Moreover, among the deduced 69 BnACTIN proteins, the molecular weight (MW) estimates range from a low of 30.28 kDa to a high of 112.57 kDa. Concurrently, their hydropathy (GRAVY) indices and isoelectric points (pIs) extend from −0.74 to 0.078 and from 4.64 to 9.35, respectively (Supplementary Table S1). Additionally, 54 out of the 69 BnACTIN proteins exhibited an instability index below 39, categorizing them as stable proteins. Furthermore, subcellular localization predictions indicated that the majority of BnACTINs, 53 in total, were cytoplasm, while ten were nuclear, five were associated with the cell membrane, and the remaining one was chloroplast. The identification of 31 *BraACTIN*s in *B. rapa* as well as 37 *BolACTIN*s in *B. oleracea* was pursued to understand the evolutionary interplay among gene family members in Brassicaceae crops, following the above consistent pipeline (Supplementary Table S2).

### 2.2. Chromosomal Localization and Phylogenetic Relationships Insights into BnACTINs

**Figure 1:**
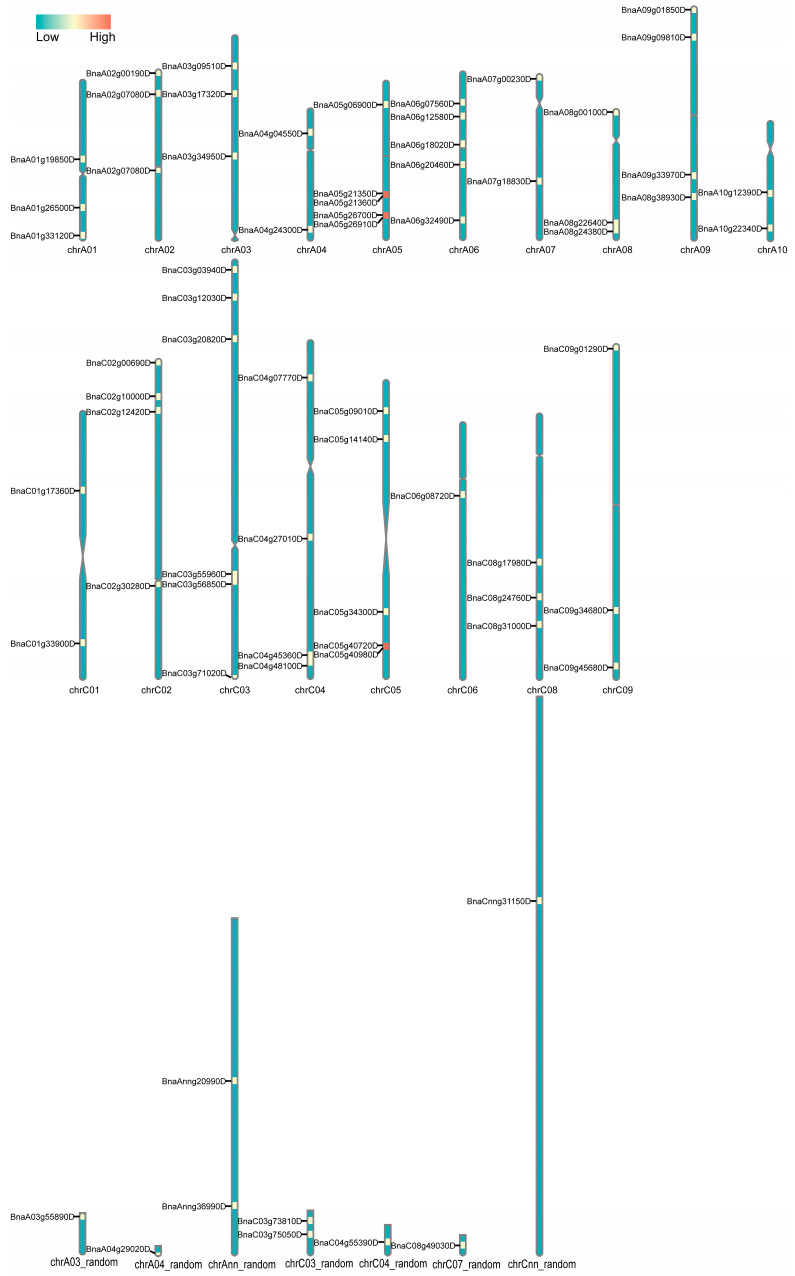
Chromosomal distribution of *BnACTIN*s. The genomic positioning of *BnACTIN*s is depicted, taking into account the centromere positions, chromosome lengths, and gene placements. The chromosome identifiers are indicated at the base of each graphical representation. An accompanying heatmap illustrates the concentration of genes per megabase across the chromosomes.

**Figure 2:**
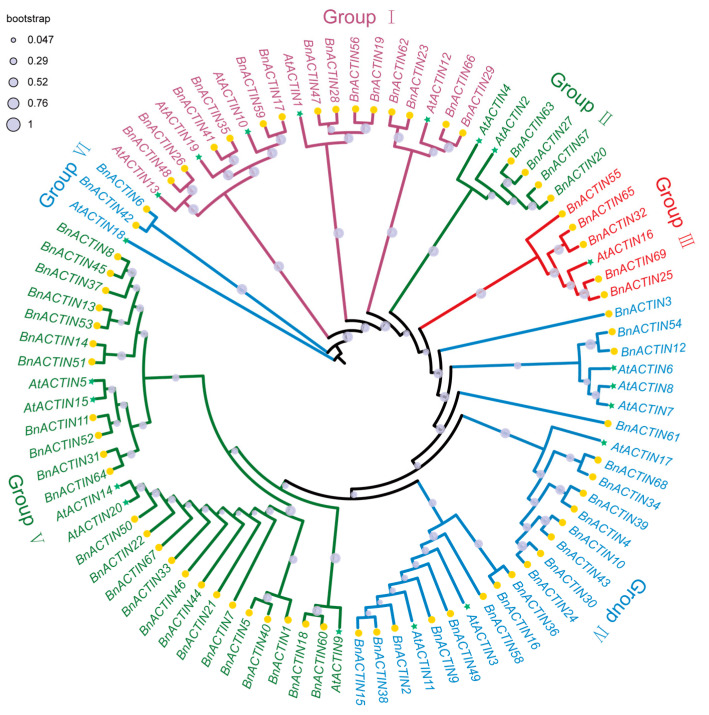
The evolutionary ties between *ACTIN* genes in *Arabidopsis thaliana* and *Brassica napus* were elucidated. A phylogenetic tree was assembled using MEGA7.0, applying the neighbor-joining technique with 1000 bootstrap iterations. The diversely colored subgroups were identified based on their positions at nodes and branches, as well as the tree’s inherent features.

The visual representation of the chromosomal locations for detected *BnACTIN*s was conducted based on their physical positions (Figure 1). The 69 *BnACTIN* genes exhibited an uneven distribution across the 19 chromosomes, with 36 genes in the A_n_ subgenome and 33 in the C_n_ subgenome (Figure 1). Every chromosome possessed at least a single ACTIN gene. On average, the A_n_ subgenome contained 3.6 *BnACTIN* genes per chromosome across its ten chromosomes, with chromosomes A07 and A10 having the fewest (2) and chromosomes A05 and A06 having the highest count (5 each). In the C_n_ subgenome, the mean count of *BnACTIN*s on each chromosome was 3.7, reaching a minimum of one on chromosome C07_random and peaking on chromosome C03. Therefore, the examination yielded no indication of a biased distribution between the two subgenomes. Additionally, *BnACTIN*s showed an uneven distribution landscape across all chromosomes. The phylogenetic relationships of *BnACTIN* genes were established by constructing a phylogenetic tree with the NJ method, integrating ACTIN proteins from *A. thaliana* [22]. 69 *BnACTINs* were categorized into six groups (Ⅰ–Ⅵ), predicted by their homologous relationship with *AtACTIN*s (Figure 2). Among the six clusters, there was a marked variation in the number of genes, with cluster Ⅴ containing the highest (24), while cluster Ⅵ the lowest (2).

### 2.3. Deciphering the Molecular Architecture of BnACTINs: Gene Structure, Motifs, and Cis-Acting Regulatory Elements

**Figure 3:**
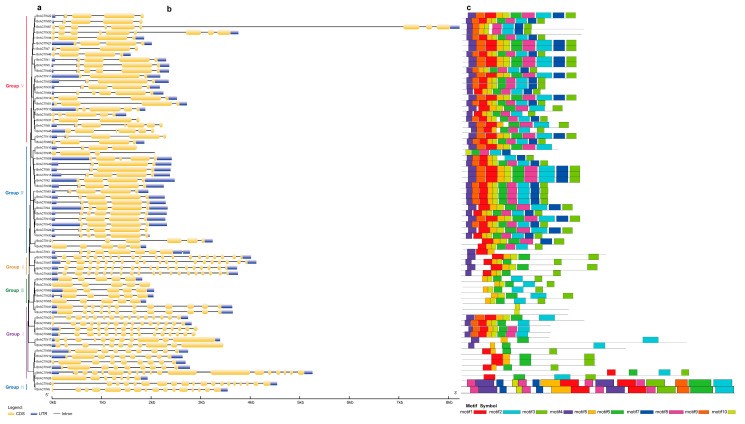
The structural composition and conserved motif patterns of BnACTINs were scrutinized in light of their evolutionary relationships. (**a**) A phylogenetic study was performed. (**b**) The gene structures of *BnACTIN*s were illustrated, using yellow for CDS and blue for UTR, with introns marked by black lines. (**c**) A MEME analysis was conducted to uncover conserved motifs in the BnACTIN protein family.

To further elucidate the evolutionary traits of *BnACTIN*s, we conducted a comparison of gene structures, conserved motifs, and *cis*-acting regulatory elements across the six clades. To investigate the diversity in gene structure among various groups of *BnACTIN*s, a comparison of their intron-exon structures was made in the context of the phylogenetic relationships (Figure 3a, Supplementary Table S3). Each *BnACTIN* was multiexon, which harbored multiple introns, especially the genes in Group Ⅱ (*BnACTIN20*, *BnACTIN27*, *BnACTIN57*, *BnACTIN63*) possessed the greatest number of exons and introns, while members in Group Ⅳ contained a relatively low number of exons (Figure 3b). Furthermore, the number of exons in *BnACTIN*s exhibited considerable variation among different subfamilies, with counts ranging from 3 to 20. Also, inaccurate annotation led to the absence of untranslated regions (UTRs) at both or one end in 28 of 69 *BnACTIN*s. Nonetheless, members within the same subfamily commonly displayed similar intron-exon organization patterns (Figure 3b). For instance, genes in Group Ⅱ typically had two exons, in contrast to Group Ⅲ, which generally contained five exons. Consequently, the distribution of introns and exons offered crucial evidence for understanding the phylogenetic connections among the members of this gene family. In addition, a total of 10 conserved motifs were discovered within the 69 *BnACTIN*s, visualized in Figure 3c. Ranging in length from 21 to 50 aa, the ten conserved motifs were dominated by the highest occurrence of motifs 5 and 6. To explore the potential functional characteristics of motifs, their sequences were searched in InterPro and CDD databases. All the motifs showed notable matches to the ACTIN domain (pfam00022) upon searching against the CDD database. Furthermore, motifs 1–8 exhibited a significant hit with ATPase, which is a nucleotide-binding domain, according to the search in the InterPro database. Distinct subfamilies exhibited variation in motif composition, yet a conserved pattern of motif distribution within the same subcategory underscored their phylogenetic ties and potential functions. Also, the examination of BnACTIN proteins led to the detection and refinement of three key structural properties (Supplementary Figure S1).

As is well-known, the presence of *Cis*-elements in the promoter regions can modulate the gene expression [41,42]. Hence, the promoters of *BnACTIN*s were investigated utilizing PlantCARE (Supplementary Table S4) [43]. In total, 93 functional *cis*-elements were found within the promoter regions of *BnACTIN*s (Supplementary Figure S2, Supplementary Table S5). Moreover, all *BnACTIN*s exhibited the presence of either TATA-box or CAAT-box elements, standard components of eukaryotic promoters. Moreover, many of these elements were associated with light response and abscisic acid response and were essential for anaerobic induction. MYC element existed in promoter regions of 66 *BnACTIN*s, which have noteworthy impacts on plant growth, seed yield, and protein content development [44]. MYB elements, which were detected in 65 of 69 *BnACTIN*s, are widely present in higher plants and are pivotal in the stress resistance response [45]. The ARE element, which is a *cis*-acting regulatory element essential for anaerobic induction, was also identified within 65 of 69 *BnACTIN*s. Furthermore, multiple *BnACTIN* promoter regions possessed elements related to light response, including Box 4 (a component of a preserved DNA unit responsible for light sensitivity), GT1-motif (light responsive element), G-box (*cis*-acting regulatory element involved in light responsiveness), TCT-motif (element of a light-reactive motif). In addition, the presence of stress-related elements, such as MBS (MYB binding site involved in drought-inducibility), ABRE (*cis*-acting element involved in the abscisic acid responsiveness) and TGACG-motif (*cis*-acting regulatory element involved in the MeJA-responsiveness), were also detected in *BnACTIN*s promoter regions, implying a significant role for *BnACTIN*s in plant development, growth, and stress responses (Supplementary Figure S2, Supplementary Table S5).

### 2.4. Syntenic Relationships and Gene Duplication of ACTIN Families

**Figure 4:**
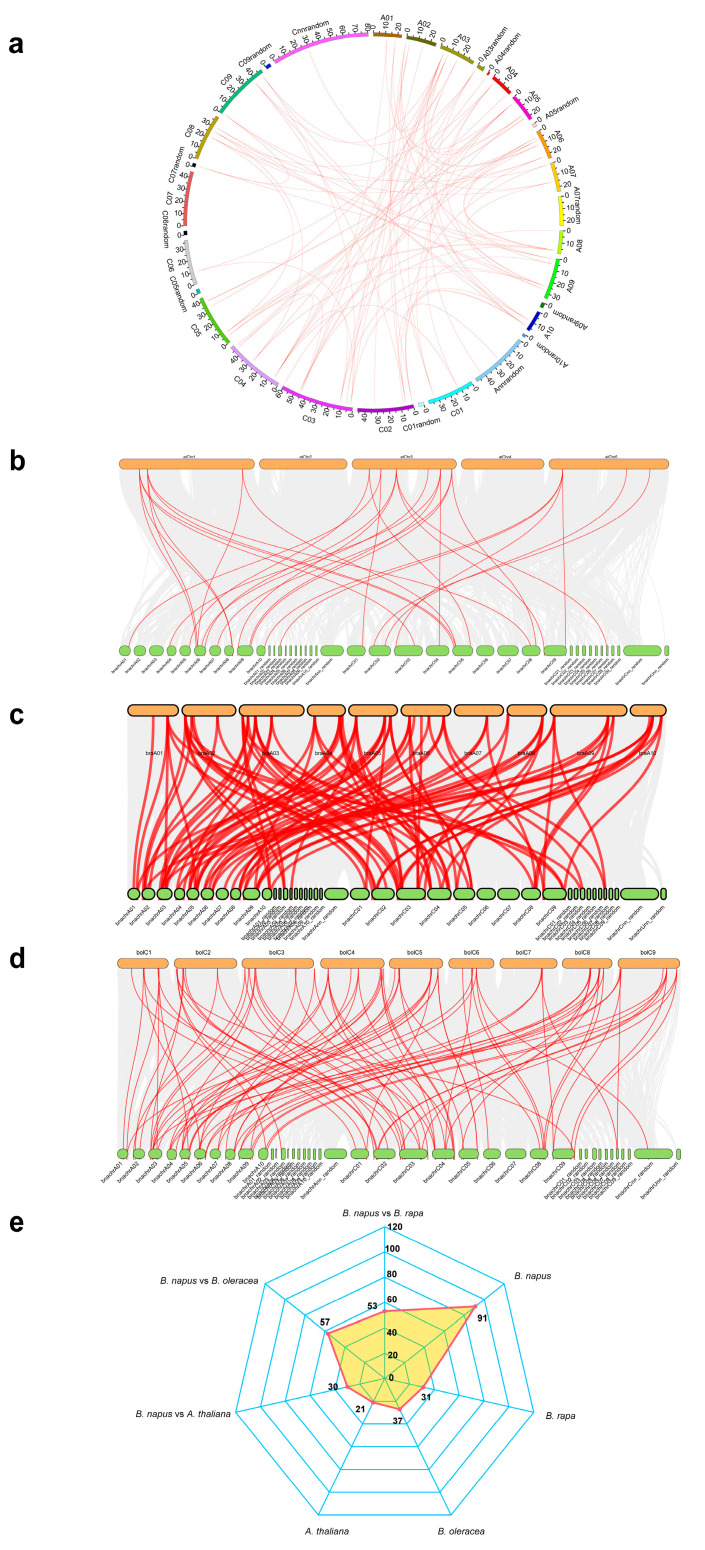
Genome-wide synteny analysis of *ACTIN* genes among *A. thaliana*, *B. napus*, *B. rapa,* and *B. oleracea* (**a**) Collinearity among the *BnACTINs*. Each chromosome of *B. napus* was indicated with different colors. The red lines represented the collinearity. (**b**) Synteny analysis of *ACTIN* genes between *A. thaliana* and *B. napus*. The gray lines indicate the whole genome collinear blocks between species, while the red lines emphasize syntenic *ACTIN* orthologs. The top orange rounded rectangle represents the *A. thaliana* chromosome, while the bottom green rounded rectangle represents the *B. napus* chromosome. (**c**) Synteny analysis of *ACTIN* genes between *B. rapa* and *B. napus*. The gray lines indicate the whole genome collinear blocks between species, while the red lines emphasize syntenic *ACTIN* orthologs. The top orange rounded rectangle represents the *B. rapa* chromosome, while the bottom green rounded rectangle represents the *B. napus* chromosome. (**d**) Synteny analysis of *ACTIN* genes between *B. oleracea* and *B. napus*. The gray lines indicate the whole genome collinear blocks between species, while the red lines emphasize syntenic *ACTIN* orthologs. The top orange rounded rectangle represents the *B. oleracea* chromosome, while the bottom green rounded rectangle represents the *B. napus* chromosome. (**e**) Radar charts showed the number of *ACTIN*s orthologous and paralogous gene pairs across four Brassicaceae species.

Syntenic analysis was conducted on the protein sequences of detected *ACTIN* genes across the three species to assess the impact of polyploidization events on the evolution of *Brassica* genomes and the *ACTIN* gene family. From the results, 91, 21, 37, and 37 paralogous *ACTIN* gene pairs were detected within *B. napus*, *A. thaliana*, *B. oleracea* and *B. rapa*, respectively (Figure 4a,e). Among the 91 paralogous *ACTIN* pairs in *B. napus*, 19 pairs were found located in the A_n_ subgenome and 11 in the C_n_ subgenome, with the remaining 61 pairs spanning both two subgenomes. These paralogous gene pairs were categorized into five distinct types: proximal, tandem, WGD (Whole Genome Duplication), dispersed, and transposed (Table 1, Supplementary Table S6). 67 *BnACTIN*s, constituting 97.1% of the total, originated from gene duplication events, driven primarily by WGD (87.9%) and transposition events (12.1%), being the key factors in the family’s enlargement (Table 1, Supplementary Table S6). With regard to the other two *Brassica* species, the progenitors of *B. napus*, *ACTIN* gene family expansion is predominantly driven by the process of WGD as well (Table 1, Supplementary Table S6).

**Figure 5:**
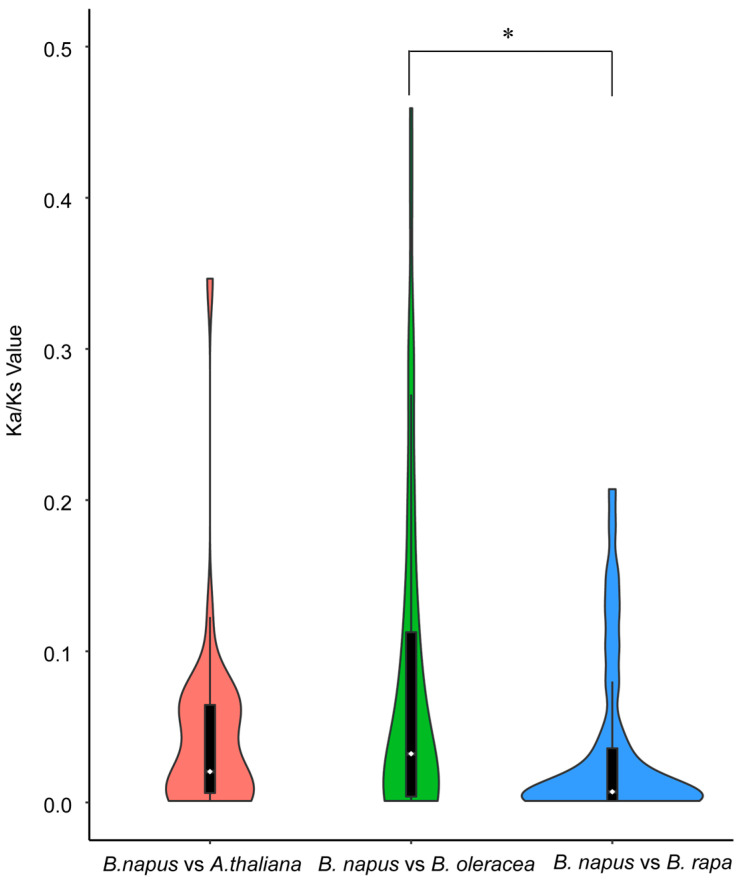
Violin plot of the pairwise Ka/Ks ratios among orthologous genes. Only orthologous gene pairs with Ks < 1 were considered. Wilcoxon Rank-Sum Tests were performed to identify significant differences among different categories of orthologous gene pairs, marked by an asterisk for *p* values less than 0.05. The violin plots feature dotted lines representing the lower, median, and upper quartiles. Comparisons between different species were presented in respective colors.

Syntenic genes, which are orthologous, are situated in syntenic fragments across different species and trace back to a common ancestor. The synteny analysis revealed that a total of 62 *BnACTIN*s exhibited collinearity in homoeologous genomic regions with their ancestors (*A. thaliana*, *B. rapa,* and *B. oleracea*). Between *B. napus* and *B. oleracea*, 57 orthologous gene pairs were discovered, with 53 identified between *B. napus* and *B. rapa* and 30 between *B. napus* and *A. thaliana* (Figure 4b–d, Supplementary Table S7). The majority of *BnACTIN*s could trace their lineage back to their ancestors. After diverging from *A. thaliana*, the *Brassica* genomes were subjected to an additional round of whole-genome triplication, so in the absence of gene loss, a solitary *A. thaliana* gene would be represented by six copies in *B. napus* as well as three copies in *B. oleracea* and *B. rapa*. [46]. According to the results, no *AtUBC* was inherited as whole six copies in *B. napus*, whereas a total of six *AtUBCs* retained three whole copies in *B. rapa* and *B. oleracea*, indicating that considerable gene loss happened as a part of the polyploid formation (Supplementary Table S7). Additionally, assessing the selection pressure on *BnACTIN*s involved computing the Ka/Ks ratios of *BnACTIN*s paralogous gene pairs as well as their orthologous counterparts to *BraACTIN*s, *BolACTIN*s, and *AtACTIN*s. Typically, a Ka/Ks ratio exceeding one indicates positive selection, a ratio of exactly one suggests neutral selection, and a ratio less than one denotes purifying selection [47]. Among *BnACTIN* paralogous gene pairs, all Ka/Ks ratios were below one, indicating that these genes were under the influence of robust purifying pressures (Supplementary Table S8). The examination of the Ka/Ks ratios for all orthologous gene pairs revealed a range of 0.001 to 0.818, with a mean of 0.070, suggesting the action of purifying selection (Supplementary Table S9). Orthologous gene pairs between *A. thaliana* and *B. napus* are estimated to have diverged approximately 15.2 million years ago, which was consistent with previous reports [48]. As *B. napus* and its progenitors, the estimation of selection pressure for their orthologous gene pair was also conducted (Supplementary Table S9). In addition, the values of the Ka/Ks ratio for orthologs between *B. napus* and *B. oleracea* were notably greater than those between *B. napus* and its other progenitor, indicating that *BnACTIN*s inherited from *B. rapa* underwent heavier purifying selection after the formation of allotetraploid *B. napus* (Figure 5).

### 2.5. Predicting Protein Interactions Network and Potential Function of BnACTIN Proteins

**Figure 6:**
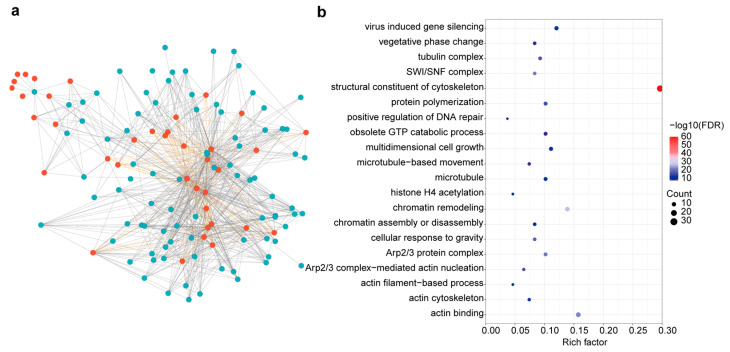
The protein interaction network analysis of ACTIN proteins. (**a**) The BnACTIN PPI network is visualized, where red circles denote BnACTIN proteins and blue circles represent their interacting proteins. Interactions with BnACTINs are marked by grey lines and self-interactions among BnACTINs by yellow lines. (**b**) GO enrichment analysis is performed to assess the functional enrichment of proteins interacting with BnACTINs.

To investigate the potential molecular mechanisms and functions of BnACTINs, predicted interaction networks were forecasted based on established protein interactions in *A. thaliana*. According to the data from the STRING database, 20 AtACTIN proteins interacted with 77 proteins in *A. thaliana*, which matched 108 orthologs in *B. napus* (Figure 6a, Supplementary Table S10). BnACTINs could interact with other family members, leading to the formation of heterodimers for involvement in a range of biological processes (Figure 6a,b). Moreover, gene ontology (GO) enrichment analysis was conducted to explore the functional roles of BnACTINs interacted proteins. The results demonstrated that BnACTINs interacted proteins were significantly enriched in a structural constituent of the cytoskeleton, chromatin remodeling, actin binding [49], cellular component (SWI/SNF complex [50], Arp2/3 protein complex [51,52]), cellular response to gravity and vegetative phase change (Figure 6b, Supplementary Table S11). Therefore, our analysis results suggested that BnACTINs might play an important role in plant development and stress response via interacting with proteins associated with these functions.

Additionally, GO enrichment analysis among *BnACTIN*s was conducted to investigate their potential biological functions. The Gene Ontology (GO) terms were categorized into three main groups: molecular functions (MF), biological processes (BP), and cellular components (CC). The functions of most *BnACTIN*s were significantly enriched in the biological processes category, including proteasomal protein catabolic process, actin cytoskeleton organization, cytoskeleton organization, and so on (Supplementary Figure S3, Supplementary Table S12). Furthermore, the remaining GO terms belonging to biological processes showed a broad relationship with plant vegetative development (vegetative phase change, root hair cell tip growth) as well as stress-induced reactions (response to freezing, response to red light). Moreover, GO cellular component (CC) enrichment (cytoskeleton, Arp2/3 protein complex) highlighted that *BnACTIN*s were predominantly located in the cytoplasm. Furthermore, GO terms structural constituent of the cytoskeleton and glycerol−3−phosphate O−acyltransferase activity indicated the molecular functions of *BnACTIN*s. The findings from the GO enrichment analysis demonstrate that *BnACTIN*s are involved in vegetative growth, cytoskeletal function, and stress response in accordance with earlier studies [11,15,49].

### 2.6. Insights into BnACTIN Gene Expression Profiling across Different Tissues and Stress Conditions

**Figure 7:**
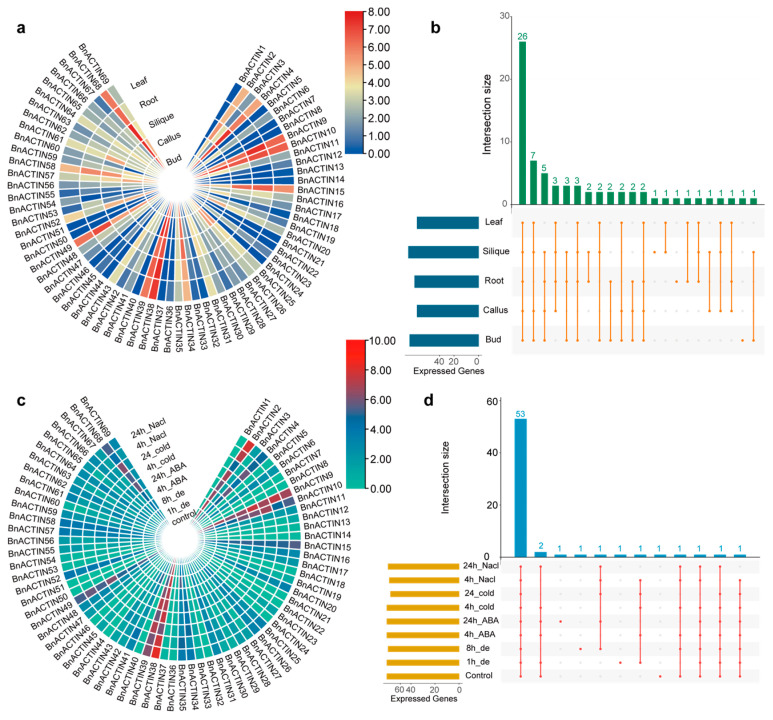
Expression profiles of *BnACTIN*s across various tissues and in response to diverse abiotic stressors. (**a**) Expression values of *BnACTIN*s across several tissues were treated using log2 transformation for normalization, and a color spectrum was utilized to represent different expression intensities. (**b**) The number of *BnACTIN*s with detectable expression in a variety of tissues is enumerated. (**c**) Expression values of *BnACTIN*s in response to multiple abiotic stress were treated using log2 transformation for normalization, and a color spectrum was utilized to represent different expression intensities. (**d**) The number of *BnACTIN*s with detectable expression in a variety of conditions is enumerated.

To explore the potential biological functions and expression patterns of *BnACTIN*s, five tissues (root, leaf, callus, bud, and silique) available RNA-seq data were collected to analyze [53]. In total, 26 *BnACTIN*s were expressed in all the above five tissues, whereas four *BnACTIN*s exhibited expression silence in any tissue (Figure 7b, Supplementary Table S13). Among different tissues, *BnACTIN*s exhibited relatively lower expression levels in the leaf (Figure 7a, Supplementary Table S13). The tissues bud and callus shared analogous expression profiles for the *BnACTIN*s. Moreover, organ-specific expressed *BnACTIN*s showed relatively weak expression levels (FPKM < 1). Some *BnACTIN*s presented tissue-preferential expression patterns, such as *BnACTIN*9, expressed highest in the root, and *BnACTIN4* was observed to be highly expressed in both silique and callus (Figure 7a, Supplementary Table S13). In addition, *BnACTIN10* (ortholog of *AT5G09810*) showed high expression levels across all tissues, suggesting its crucial involvement in plant growth processes.

Beyond examining the expression patterns across various tissues, the expression profiles of *BnACTIN*s under various stress conditions were investigated as well. The RNA-seq data of samples under several abiotic treatments (ABA, cold, dehydration, and salinity) were obtained from public studies [54]. In response to various abiotic stresses, the expression levels of the majority of *BnACTIN*s were up-regulated, excluding those expressing silent or weak genes (Figure 7c, Supplementary Table S14). Moreover, a total of 53 *BnACTIN*s were expressed under all stress conditions (Figure 7d). Notably, the expression levels of *BnACTIN35* increased under all treatments, and a subset of genes (*BnACTIN2*, *BnACTIN9*, *BnACTIN35*, *BnACTIN38*, *BnACTIN55*) displayed enhanced expression in response to NaCl stress. Strikingly, *BnACTIN64* was solitary among *BnACTIN*s that experienced expression down-regulation under each abiotic stress condition.

### 2.7. Genetic Effects of BnACTIN Genes on Agronomic Traits

In order to examine the genetic diversity of *BnACTIN*s, SNPs were detected in a global natural population comprising 324 accessions (Supplementary Table S15) [55]. In total, 3096 SNPs were identified in *BnACTIN*s, with each gene possessing 48 SNPs on average, which exceeded the whole genome-wide frequency (36 SNPs per gene). According to the annotation analysis, among SNPs in *B. napus*, 1080 (10.637%) out of them were located in exons, and 1097 SNPs comprising 198 missense mutations, six nonsense mutations, and 893 stop codon-causing mutations, led to the diversity of amino acid sequences (Supplementary Table S16). The results revealed that *BnACTIN*s in the A_n_ subgenome contained an average of 60 SNPs per gene, which was higher than that in the C_n_ subgenome (36). Moreover, the number of SNPs within different groups ranges from 92 (Group Ⅱ) to 33 (Group Ⅴ), suggesting SNP density varied widely among subgroups. Furthermore, discrepancies in SNP counts were noted among certain paralogous pairs of *BnACTIN*s, such as *BnACTIN33* containing 105 SNPs in contrast to its paralog *BnACTIN40* possessing only 10 SNPs. In addition, the paired *t*-test uncovered the significant variation in SNP density between *BnACTIN* paralogous gene pairs.

**Figure 8:**
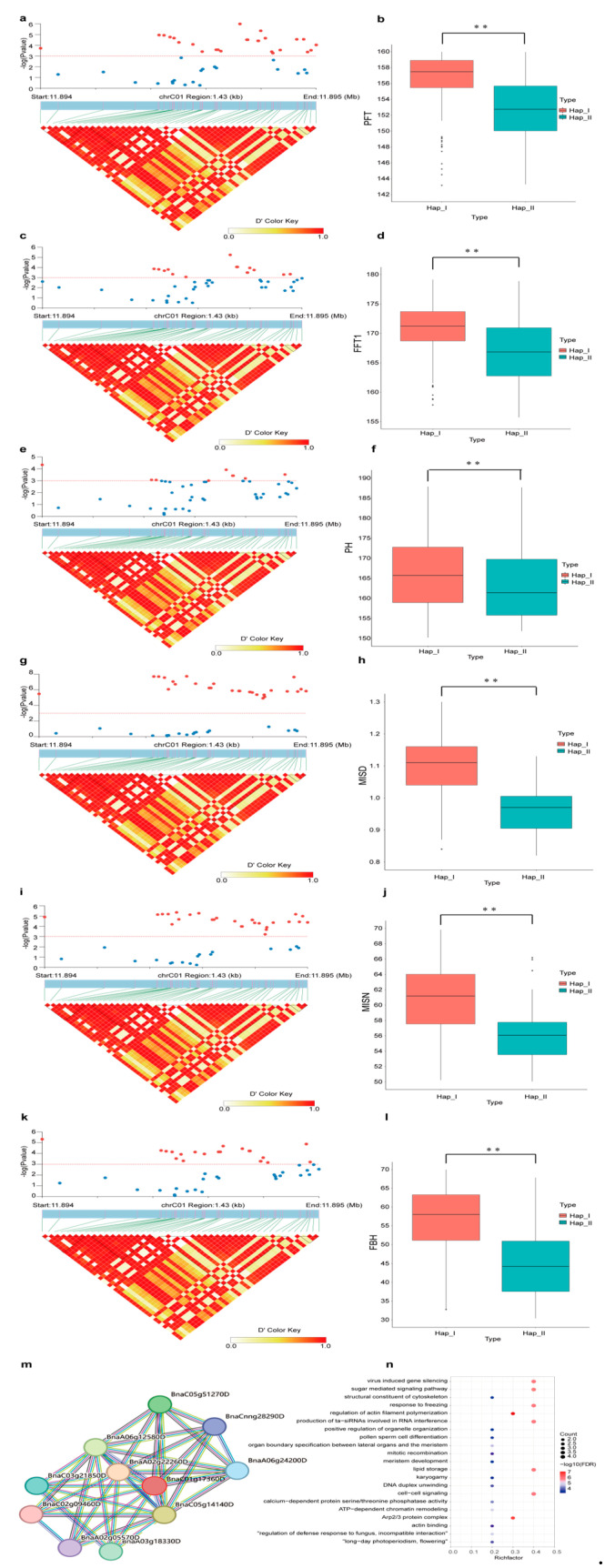
Whole-genome association mapping analysis of *BnACTIN37* in *B. napus* germplasm with 324 core collections. (**a**) Significant association of *BnACTIN37* with primary flowering time. (**b**) The box plot exhibited a primary flowering time trait comparison between two haplotypes divided based on the most significantly associated SNP in *BnACTIN37.* (**c**) Significant association of *BnACTIN37* with full flowering time. (**d**) The box plot exhibited a full flowering time trait comparison between two haplotypes divided based on the most significantly associated SNP in *BnACTIN37*. (**e**) Significant association of *BnACTIN37* with plant height. (**f**) The box plot exhibited plant height trait comparison between two haplotypes divided based on the most significantly associated SNP in *BnACTIN37*. (**g**) Significant association of *BnACTIN37* with main inflorescence silique density. (**h**) The box plot exhibited the main inflorescence silique density trait comparison between two haplotypes divided based on the most significantly associated SNP in *BnACTIN37*. (**i**) Significant association of *BnACTIN37* with main inflorescence silique number. (**j**) The box plot exhibited the main inflorescence silique number trait comparison between two haplotypes divided based on the most significantly associated SNP in *BnACTIN37*. (**k**) Significant association of *BnACTIN37* with first branch height. (**l**) The box plot exhibited the first branch height trait comparison between two haplotypes divided based on the most significantly associated SNP in *BnACTIN37*. (**m**) Protein–protein interaction network of BnACTIN37. (**n**) GO enrichment analysis of proteins interacted with BnACTIN37. ** indicated extremely significance (*p*-value < 0.01).

In plants, ACTINs are crucial for numerous organismal development and growth, routine cellular processes, which may finally affect phenotype [11,56,57]. Genome-wide association mapping analysis was conducted on several selected key agronomic characteristics to assess the impact of *BnACTIN*s on agronomic traits. A total of 310 SNPs across four *BnACTIN*s showed significant associations with the examined agronomic traits (Table 2, Supplementary Table S15) (*p-*value < 0.001). Remarkably, *BnACTIN37* was significantly associated with most agronomic traits, including primary flowering time, full flowering time, plant height, main inflorescence silique density, main inflorescence silique number, and first branch height (Figure 8a–i). The *t*-test between the two genotypes categorized based on the most strongly linked SNP in *BnaACTIN37* verified their significant diversity of traits. The STRING platform was utilized to acquire the interaction network of the BnACTIN37 [58] (Figure 8m), and its interacted proteins were notably enriched in functions such as regulation of actin filament polymerization, Arp2/3 protein complex, cell-cell signaling, response to freezing and so on (Figure 8n).

*BnACTIN10* was significantly associated with metabolites like oleic acid, eicosenoic acid, and erucic acid (Supplementary Figure S4e–j). Moreover, acquiring the protein interaction network for BnACTIN10 was also achieved through STRING (Supplementary Figure S5a), and its interacted proteins were enriched in functions such as regulation of actin filament polymerization, Arp2/3 protein complex, cell-cell signaling, response to freezing, and so on (Supplementary Figure S5b). Additionally, *BnACTIN29* was notably associated with stearic acid and oleic acid (Supplementary Figure S4a–d), and *BnACTIN36* was significantly associated with erucic acid (Supplementary Figure S4k,l).

## 3. Discussion

Actin is the most abundant protein in the cytoplasm of eukaryotic cells, whose amino acid sequence is highly conserved. In plant cells, the actin cytoskeleton constitutes a highly dynamic and responsive network to stimuli, playing a role in vital functions across numerous developmental and growth-related cellular activities [15,16]. The actin-involved cellular process is primarily facilitated by the collective actions of the actin multigene family, the members of which exhibited tissue-biased expression profiles. The *actin* gene family has been explored whole genome-wide in numerous plants such as *A. thaliana* [22], sweet potato [24], rice [22], grape [23], and populus [4]. Nevertheless, extensive research on the *ACTIN* gene family in *B. napus* is still lacking. Allotetraploid *B. napus* serves as an ideal subject for research into the genetic implications of polyploidy. Our research facilitates the investigation of the functions and evolution process of homologs after polyploid formation.

Totally, 69 *actin genes* were detected in *B. napus,* which uncovered that the quantity of *ACTIN* genes (20 in *A. thaliana*, 16 in grape, 22 in rice, 18 in poplar, and 30 in sweet potato, 69 in *B. napus*) does not clearly mirror the extent of the genome (~125, ~470, ~900, ~380, ~785, ~825 Mb). Post-divergence from *Arabidopsis*, the *Brassica* genus underwent a genome triplication. Later, allotetraploid species *B. napus* originated from a natural cross of *B. rapa* and *B. oleracea* approximately 7000 years ago [32]. Therefore, it is anticipated that Each A. thaliana gene has a counterpart in the form of six copies within *B. napus* due to the occurrence of two whole-genome duplication events [59]. Nevertheless, only 69 actin genes were discovered in *B. napus*, which was nearly 3.5 times superior in quantity to *AtACTIN*s, and the count of *BraACTIN* and *BolACTIN* genes was also under three times of that in *A. thaliana*. Remarkably, all *AtACTIN*s retained incomplete copies in *B. napus.* The findings suggest that the phenomenon of gene loss took place following the originating of the *Brassica* and allotetraploid *B. napus*. Gene duplication in higher eukaryotes, including whole genome duplication (WGD), tandem duplication, and chromosomal segmental duplication, is recognized as a primary driver of gene family enlargement [60,61,62]. Collinearity analysis among paralogs reveals WGD provides a predominant force in genome expansion for *Brassica* species, consistent with previous studies [63,64,65] (Table 1). In addition, with all paralogous *BnACTIN* gene pairs exhibiting a Ka/Ks value below one, it demonstrates the action of purifying selection in the evolutionary process of *BnACTIN*s (Supplementary Table S8).

*BnACTIN*s were categorized into six clusters based on the phylogenetic relationship along with *AtACTIN*s, corresponding to the insights of earlier research [11] (Figure 2). Additionally, the phylogenetic categorization of *BnACTIN*s received additional validation through the examination of gene architecture and the conservation of the motif. All the *BnACTIN*s were discovered to contain the motifs aligning with the actin domain, and ATPase-like associated motifs were also detected, implying their roles in ATPase activity. Moreover, the results display that the members within the same subfamily frequently exhibited a consistent intron-exon and conserved motif arrangement. However, among the various subfamilies, the exon numbers in *BnACTIN*s showed notable diversity, suggesting diverse morphological characteristics of *BnACTIN*s. For instance, genes in Group Ⅱ harbored 20 exons, while those in Group Ⅲ possessed only five (Figure 3). Residing in promoter areas, *cis*-acting elements are responsible for the regulation of gene transcription involved in physiological processes like abiotic stress responses, plant growth, and development, establishing foundational functional connections within intricate regulatory networks [66,67]. The TATA-box and CAAT-box are common in eukaryotic organisms, which form the binding sites for RNA transcription factors to regulate the gene transcription exhibited in all *BnACTIN*s (Supplementary Figure S2). Apart from these ubiquitous elements, others associated with stress adaptation (MYB, MBS, ABRE, TGACG-motif), light responsive (Box 4, GT1-motif, G-box, TCT-motif), plant growth (MYC) as well as anaerobic induction (ARE) were likewise located across *BnACTIN*s, implying their essential functions in plant development, reactions to abiotic stress and light. Unusually, acting regulatory elements varied among the members in identical clusters, denoting the possibility of their diverse roles [68,69].

A preponderance of BnACTINs was detected to be located in the cytoplasm, which confirms their roles in the formation of the actin cytoskeleton [16] (Supplementary Table S1). Furthermore, the insights from GO enrichment analysis propose that *BnACTIN*s are anticipated to participate primarily in cytoskeleton organization, stress response, cellular process, and so on (Supplementary Figure S3, Supplementary Table S12). In spite of the functional prediction, detailed functional verification of *BnACTIN*s was previously absent. Publicly available RNA-Seq data were facilitated to investigate the expression profiles of *BnACTINs* across different tissues and stress conditions [53,54]. The expression patterns of *BnACTIN*s fluctuated across a range of tissues, while it presented relatively lower in the leaf compared to other tissues (Figure 7a, Supplementary Table S13). Multiple genes showed tissue-specific or tissue-preferential, implying their functions involved in tissue differentiation similar to the phenomenon in *A. thaliana* [24]. *ACTIN*s are recognized for their involvement in plant abiotic stress resistance [11,70,71]. ACTIN filaments are pivotal in the signaling pathways triggered by stress, serving as either direct targets or signal transducers [12]. In the face of multiple stress factors, the majority of *BnACTIN*s altered expression levels (Figure 7c, Supplementary Table S14). Moreover, a notable alteration in the expression of *BnACTIN*s was observed under NaCL treatment, suggesting that they are especially susceptible to the effects of salinity. For instance, the expression of *BnACTIN35* was increased to 4.4 times after 4 h of cold treatment. Remarkably, the expression of *BnACTIN64* was downregulated in each stress environment, indicating that abiotic stress led to different gene expression regulation. Furthermore, the findings demonstrated that each BnACTINs is involved in a multitude of intricate protein interaction networks, and their interacting proteins participate in various cellular activities (Figure 6).

In order to expose the genetic contributions of *BnACTIN*s to agronomic characteristics, SNP within *BnACTIN*s was discovered among accessions of a *B. napus* natural population (Supplementary Table S15) [55]. With an average SNP density of 48 SNPs per gene, *BnACTIN*s showed a slightly higher frequency compared to the overall genome average of 36 SNPs per gene, suggesting a significant accumulation of polymorphisms in these genes over time. The SNP density within *BnACTIN*s of the A_n_ subgenome exceeded that of the C_n_ subgenome, revealing the asymmetric evolutionary processes of *BnACTIN*s across two subgenomes, which align with patterns observed in other *B. napus* gene families [65,72,73,74]. Except for the unbalanced SNP density between subgenomes, SNP counts within paralogs were also notably divergent in *B. napus,* implying their potential functional differentiation. Previously, some studies have explored the connection between actin genes and plant immunity [75,76], whereas our understanding of their roles in agricultural traits remains limited. According to the association mapping analysis, *BnACTIN37* was significantly associated with multiple traits, including PFT, FFT1, PH, MISD, MISN, and FBH. Additionally, *BnACTIN10* was discovered to be notably linked to several agronomic traits (OA, EA1, EA2) as well. Furthermore, the interacted proteins of these genes primarily participate in the formation of the actin cytoskeleton, Arp2/3 complex, and response to freezing. Consequently, these findings offer a precious collection of potential *BnACTIN*s that could affect agronomic traits.

## 4. Materials and Methods

### 4.1. Identification of ACTIN Genes in B. napus and Its Progenitors

The genome sequence and annotation information of *B. napus* were retrieved and downloaded from the Genoscope database (http://www.genoscope.cns.fr/brassicanapus/ accessed on 10 June 2024) [32]. The datasets of its progenitors *B. rapa* ‘Chiifu’ (v3.0) and *B. oleracea* ‘HDEM’ (broccoli) were obtained from databases (https://bigd.big.ac.cn/gwh/Assembly/134/show, accessed on 10 June 2024) [77] and (http://www.ocri-genomics.org/bolbase/index.html, accessed on 10 June 2024) [78], respectively. The annotated protein sequences were HMM searched against the queries, which was an annotation file of Actin domain (PF00022) downloaded from the Pfam database (https://www.ebi.ac.uk/interpro/, accessed on 11 June 2024) applying HMMER version 3.1 (http://hmmer.org/, accessed on 11 June 2024) [79] with E-value setting as1e–20 [80]. Then, utilizing the amino acid sequences of the BnACTINs identified earlier, BLASTP searches were performed against the complete protein sequences of 20 AtACTINs available in the TAIR database (http://www.arabidopsis.org/, accessed on 11 June 2024), applying an E-value cutoff of less than 1e–20. Moreover, the candidate *ACTIN* genes were verified by the SMART databases [81] and the NCBI Conserved Domain Database [82] based on the existence of the target Actin domain. In addition, the Actin family members were identified in *B. rapa* and *B. oleracea* using the consistent method. Furthermore, in order to predict the molecular weights (MWs) and isoelectric point (pI), as well as instability indexes of the BnACTINs, their protein sequences were subjected to the online software ProtParam (https://web.expasy.org/protparam/, accessed on 29 September 2024) [83]. CELLO v2.5 [84] predicted the subcellular location of these BnACTIN proteins.

### 4.2. Phylogenetic Analysis of BnACTIN Family Members

A phylogenetic analysis was executed to shed light on the genetic relationships within the ACTIN between Brassicaceae species. Sequence alignments between BnACTIN and AtACTIN amino acid sequences were conducted using Clustal W [85] in the MEGA program with default parameters [85]. MEGA facilitated the creation of the phylogenetic tree, applying the neighbor-joining (NJ) technique, pairwise deletion, and 1000 bootstrap replicates for robustness [86]. Furthermore, online software iTOL v6 was applied for the beautification and visualization of phylogenetic trees [87].

### 4.3. Synteny Analysis of ACTIN Gene Family and Ka/Ks Evaluation

The DupGen_finder tool (https://github.com/qiao-xin/DupGen_finder, accessed on 15 June 2024) [88] was applied to ascertain the gene duplication mechanisms for paralogous UBC genes across *A. thaliana*, *B. napus*, *B. rapa*, and *B. oleracea*. Moreover, paralogs in *BnACTIN*s located on syntenic chromosome segments were visualized through the graphical capabilities of the Circos software (https://circos.ca/software/, accessed on 29 September 2024) [89]. MCScanX [90] was applied to detect the orthologs between *B. napus* and its ancestors (*B. rapa*, *B. oleracea*, and *A. thaliana*) with the default setting. At the same time, TBtools (https://github.com/CJ-Chen/TBtools/releases, accessed on 29 September 2024) [91] were used to display duplication events among orthologs. Furthermore, the fmsb package in R software was utilized to illustrate the syntenic connections among these *ACTIN* paralogous genes. In addition, The KaKs calculator [92] was implemented to assess the divergence of nonsynonymous (Ka) and synonymous (Ks) substitution rates. The evolutionary pressure (Ka/Ks ratio) on UBC orthologous genes between *B. napus* and the trio of *A. thaliana*, *B. rapa*, and *B. oleracea* was determined based on their coding DNA sequences (CDSs). Moreover, to reduce inaccuracies, gene pairs with a Ks value exceeding one were excluded from subsequent analysis [93,94].

### 4.4. Chromosomal Mapping, Structural Study, and Motif Conservation of BnACTINs

The genomic annotation from the GENOSCOPE database (http://www.genoscope.cns.fr/brassicanapus/, accessed on 20 June 2024) delineated the chromosomal positions, coding, and protein sequences of *B. napus*. RIdeogram package in the R software (https://github.com/TickingClock1992/RIdeogram, accessed on 20 June 2024) [95] was utilized to map the physical locations of *BnACTIN*s to their corresponding chromosomes. Moreover, multiple alignments between BnACTIN protein sequences were carried out by applying CLUSTAL v2.1 using default settings. The layout of the *BnACTIN*s gene structure was diagrammatically represented through the Gene Structure Display Server 2.0 (http://gsds.cbi.pku.edu.cn/, accessed on 21 June 2024) [96]. The MEME server (http://meme-suite.org/tools/meme, accessed on 21 June 2024) was utilized to explore conserved motifs within BnACTIN proteins, employing parameters as a maximum of 10 motifs, motif width ranging from 6 to 100 amino acids, and an E-value threshold of less than 1e–10 [97].

### 4.5. Cis-Acting Regulatory Element and Protein Interaction Detection of BnACTINs

PlantCARE [43] was applied for the identification of the *cis*-elements from the 2-kb promoter region of the *BnACTIN* gene sequences. In addition, the protein–protein interaction data for ACTIN proteins in *A. thaliana* were retrieved from the STRING database [58], which was then employed to predict functional association networks of BnACTINs according to their collinear relationship. The protein–protein interaction (PPI) was visualized using Cytoscape [98]. Furthermore, the BnACTINs interacted proteins were subjected to Gene Ontology enrichment analysis using the R package clusterProfiler [99] to explore their biological roles.

### 4.6. Profiling Expression Levels and Conducting GO Enrichment Analysis for BnACTINs

The available transcriptome data from several tissues (root, leaf, callus, bud, and silique) as well as stress conditions (cold, salt, dehydration, and ABA) of *B. napus* cultivar “ZS11” were obtained from public databases (National Genomics Data Centre under the project ID: CRA001775) [53,54]. Stringtie software (https://bioinformaticshome.com/tools/rna-seq/descriptions/StringTie.html#gsc.tab=0, accessed on 29 September 2024) [100] was applied to calculate the pression levels of *BnACTIN*s following alignment with Hisat2 [101]. Additionally, the gene expression profiles visualization was performed to generate a clustered heatmap using the TBtools software [91]. Subsequently, a Gene Ontology (GO) enrichment analysis for the *BnACTIN*s was conducted employing the clusterProfiler package within the R programming.

### 4.7. Association Mapping Analysis of TPS Genes within B. napus Germplasm Collection

In an effort to explore the genetic diversity of *ACTIN* genes in *B. napus*, a worldwide collection of 324 natural accessions was employed in this study [55]. The SnpEff program [102] was facilitated to extract and annotate SNPs within *BnACTIN*s gene loci. This research investigated a range of agronomic traits such as plant height (PH), first branch height (FBH), primary flowering time (PFT) (approximately more than 30% of the buds on the open into flowers), full flowering time (FFT1) (approximately more than 70% of the buds on the plant open into flowers), main inflorescence silique density (MISD), main inflorescence silique number (MISN), thioglycoside (THI), stearic acid (SA) as well as erucic acid (EA2). Family-based association mapping analysis, which accounts for population structure and relative kinship, was conducted using the mixed linear model in EMMAX [103]. Haplotype blocks and linkage disequilibrium were graphically represented using the LDBlockShow tool [104]. In addition, the STRING database provided the interaction networks of *B. napus* proteins [105].

## 5. Conclusions

The current investigation delivers a complete examination of the *ACTIN* family within *B. napus.* In total, 69 *BnACTIN*s were detected and categorized into six clusters. Members of the same subfamily displayed analogous gene structures and shared motifs. The identification of crucial cis-acting regulatory elements and functional prediction of *BnACTIN*s demonstrate that they contributed importantly to actin cytoskeleton construction and stress response. Additionally, protein–protein interaction analysis revealed that *BnACTIN*s participate in routine cellular activities. *BnACTIN* genes exhibited diverse expression behaviors across multiple tissues and in response to various abiotic stresses. Furthermore, the association mapping also highlighted the possibility that *BnACTIN* genes could contribute to agronomic traits in *B. napus*. In conclusion, these findings have delivered a comprehensive insight into *BnACTIN* genes, offering a groundwork for advanced functional exploration and genetic enhancement in *B. napus* cultivation.

## Figures and Tables

**Table 1:** Duplication patterns of *ACTIN* paralogs across four Brassicaceae species (*B. napus*, *B. rapa*. *B. oleracea,* and *A. thaliana*).

Species	WGD	Tandem	Proximal	Transposed	Dispersed	Total
*B. napus*	80	0	0	11	0	91
*B. rapa*	24	0	0	7	0	31
*B. oleracea*	26	1		10	0	37
*A. thaliana*	4	0	1	16	0	21

**Table 2:** Overview of *BnACTIN* genes associated with important phenotypic traits in *B. napus*.

Agricultural Traits.	Significantly Associated Genes
Primary Flowering Time (PFT)	*BnACTIN37*
Full Flowering Time (FFT1)	*BnACTIN37*
Plant Height (PH)	*BnACTIN37*
Main Inflorescence Silique Density (MISD)	*BnACTIN37*
Main Inflorescence Silique Number (MISN)	*BnACTIN37*
Thioglycoside (THI)	*BnACTIN29*
Stearic Acid (SA)	*BnACTIN29*
Oleic Acid (OA)	*BnACTIN10*
Eicosenoic Acid (EA1)	*BnACTIN10*
Erucic Acid (EA2)	*BnACTIN10 BnACTIN36*
First Branch Height (FBH)	*BnACTIN37*

## Data Availability

The associated data are detailed within the Supplementary Materials.

## Supplementary Materials

The following supporting information can be downloaded at https://www.mdpi.com/article/10.3390/ijms251910752/s1.
