# Supplementary material for: Genome-Wide Profiling of the ACTIN Gene Family and Its Implications for Agronomic Traits in Brassica napus: A Bioinformatics Study
Source: Int J Mol Sci. 2024 Oct 6;25(19):10752. doi: 10.3390/ijms251910752 (PMC11476578; doi:10.3390/ijms251910752)
Supplement: Supplementary file 1 [file ijms-25-10752-s001.zip › Supplementary Figure S3.pdf]

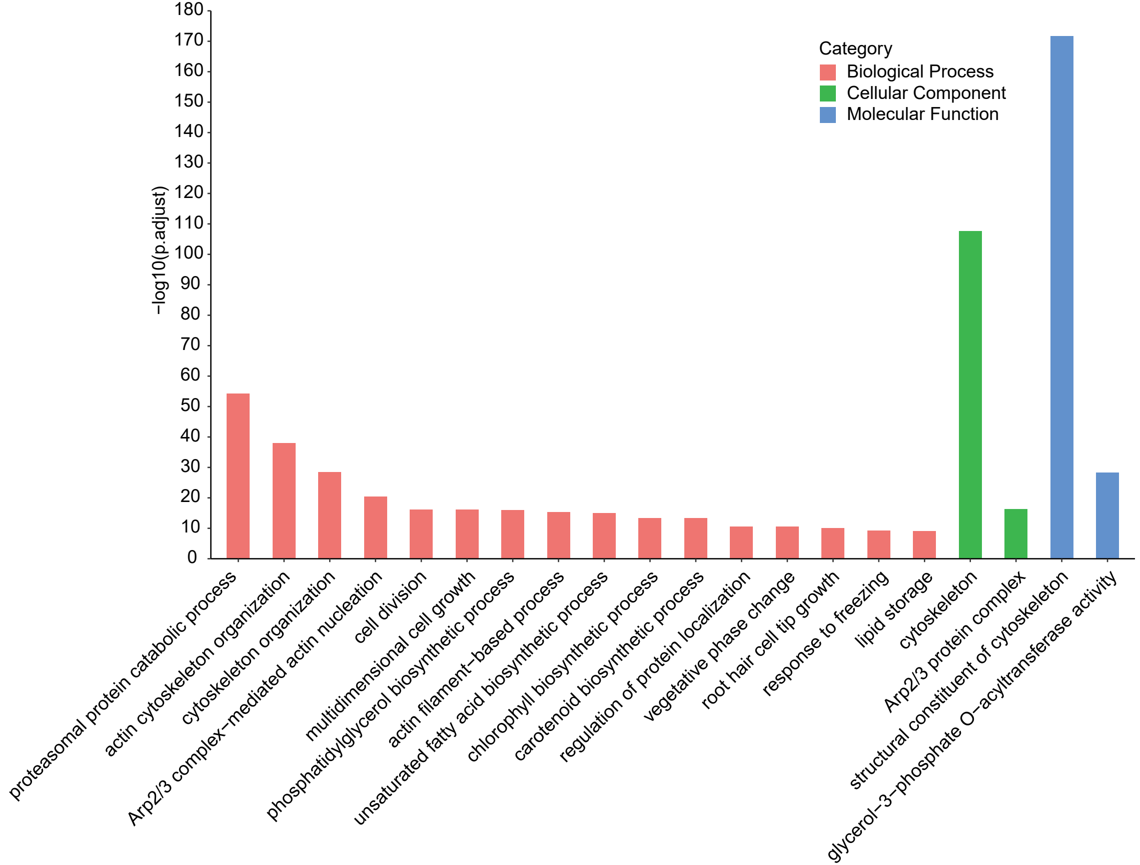

Supplementary Figure 3. GO enrichment analysis of *BnACTIN*s based on their categories: biological process (BP), molecular function (MF） and cellular component (CC).
