# Supplementary material for: Genome-Wide Profiling of the ACTIN Gene Family and Its Implications for Agronomic Traits in Brassica napus: A Bioinformatics Study
Source: Int J Mol Sci. 2024 Oct 6;25(19):10752. doi: 10.3390/ijms251910752 (PMC11476578; doi:10.3390/ijms251910752)
Supplement: Supplementary file 1 [file ijms-25-10752-s001.zip › Supplementary Figure S4.pdf]

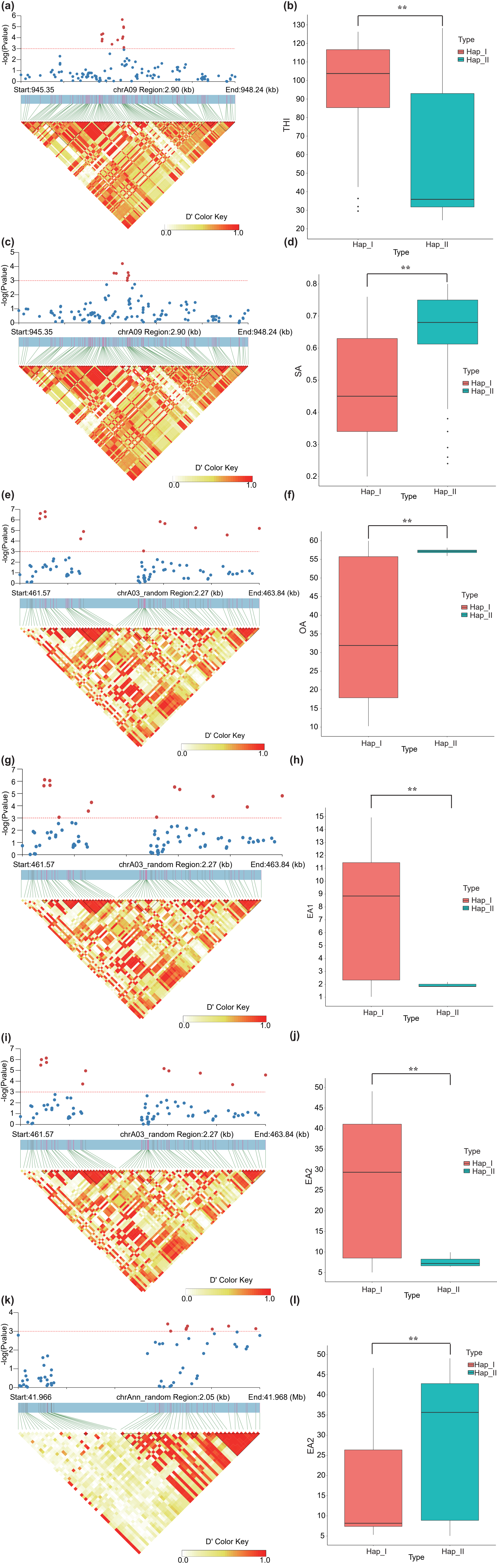

Supplementary Figure 4. Genome wide association study of *BnACTINs* in *B. napus* population. (a, b) *BnACTIN29* presenting significant association with thioglycoside. (c, d) *BnACTIN29* presenting significant association with stearic acid. (e, f) *BnACTIN10* presenting significant association with oleic acid. (g, h) *BnACTIN10* presenting significant association with eicosenoic acid. (i, j) *BnACTIN10* presenting significant association with erucic acid. (k, l) *BnACTIN36* presenting significant association with erucic acid.
