# Supplementary material for: Genome-Wide Profiling of the ACTIN Gene Family and Its Implications for Agronomic Traits in Brassica napus: A Bioinformatics Study
Source: Int J Mol Sci. 2024 Oct 6;25(19):10752. doi: 10.3390/ijms251910752 (PMC11476578; doi:10.3390/ijms251910752)
Supplement: Supplementary file 1 [file ijms-25-10752-s001.zip › Supplementary Figure S5.pdf]

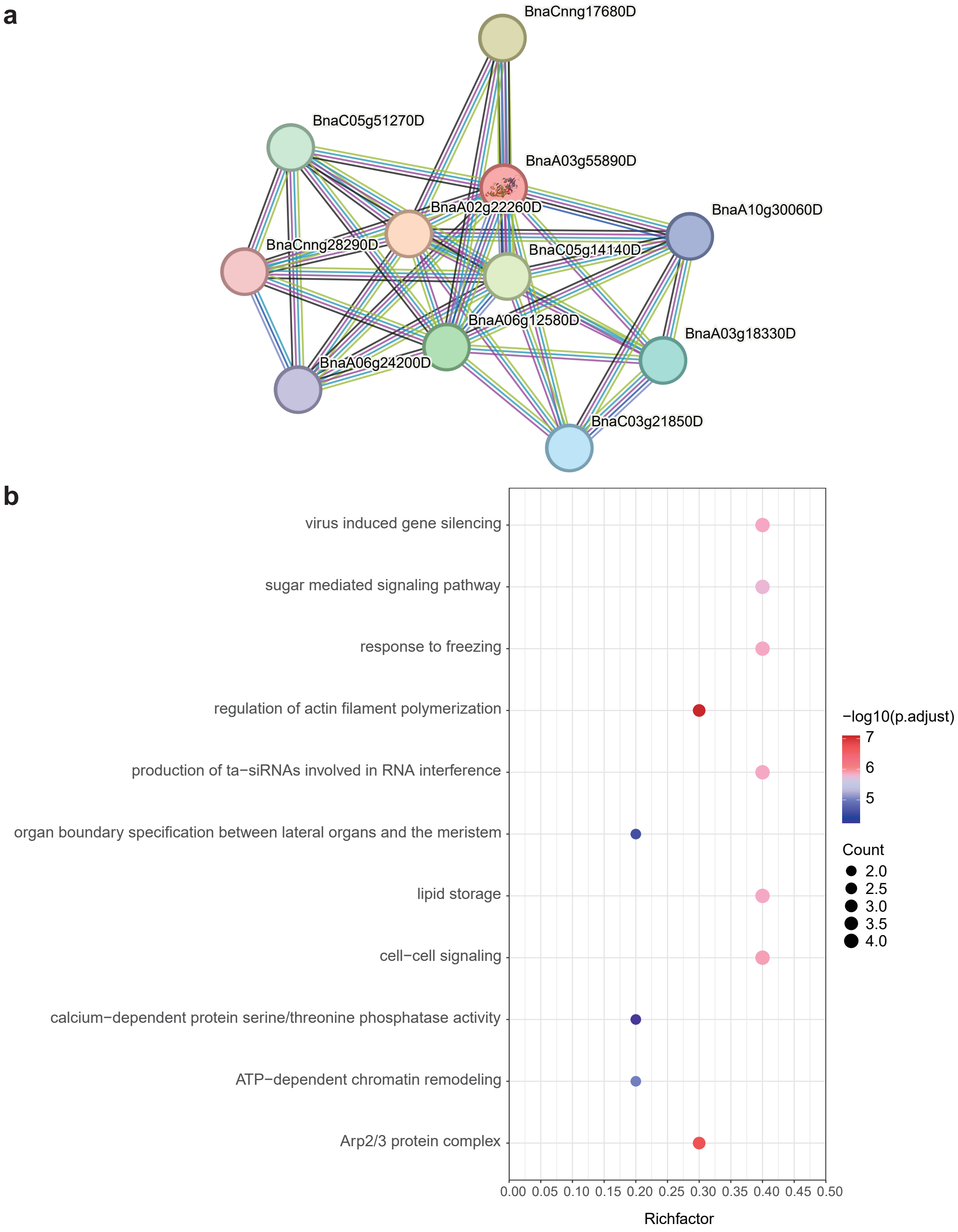


Supplementary Figure 5. Interacting partners of the BnACTIN10 protein. (A) Interaction network of BnaA09g01850D. B) GO enrichment analysis of BnaA09g01850D-interacting proteins.
